# Sapphire/silicon-substrate dependent magnetism of Er-substituted ZnO (ZnO: Er) thin film: x-ray absorption near edge structure (XANES) study

**DOI:** 10.1038/s41598-025-25017-0

**Published:** 2025-11-20

**Authors:** Shivani Das, Kuan-Hung Chen, Tapaswini Chhotaray, Wei-Xuan Lin, Surajit Ghosh, Dilip Kumar Mishra, Sekhar Chandra Ray, W. F. Pong

**Affiliations:** 1 Department of Physics, Faculty of Engineering and Technology (ITER), Siksha ‘O’ Anusandhan Deemed to be University, Bhubaneswar, 751 030 Odisha India; 2 Department of Physics, Tamkang University, Tamsui 251, Taipei, Taiwan; 3 Department of Physics, CSET, University of South Africa, Florida Science Campus, Private Bag X6, Christiaan de Wet and Pioneer Avenue, Florida Park, Florida, Johannesburg, 1710 South Africa

**Keywords:** ZnO, ZnO:Er, XANES, XPS, UPS, Materials science, Nanoscience and technology, Physics

## Abstract

Hexagonal wurtzite structure Er-substituted ZnO dilute magnetic semiconductors (DMSs) thin films have been deposited on sapphire/silicon substrate [(ZnO: Er)_sapphire/silicon_] using DC sputtering technique at room temperature in oxygen-plasma atmosphere and studied their electronic/local atomic-structure and correlated with their magnetic behaviors. Incorporation of Er^+3^-ion in ZnO-lattice [(ZnO: Er)_sapphire/silicon_] and formation of Zn^+2^/O^−^ vacancies (V_Zn_/V_0_) at the bonding between Zn and O_2_ may induces room temperature ferromagnetism. Magnetic M-H loops of pure-ZnO shows diamagnetic behaviour, whereas (ZnO: Er)_sapphire/silicon_ are ferromagnetic in nature. Moreover, (ZnO: Er)_sapphire_ is higher ferromagnetic (saturation magnetization, M_S_ ≈2.3 emu/g, coercivity, H_C_ ≈63 Oe) than (ZnO: Er)_silicon_ (M_S_ ≈0.5 emu/g, H_C_ ≈15 Oe). Incorporation of Er^+3^-ion and formation of V_Zn_/V_0_ vacancies were observed from x-ray photoelectron spectroscopy (XPS) and x-ray absorption near edge structure (XANES) spectroscopy analysis. The peak positions of Zn 3*p* and O 1*s* in XPS spectrum of (ZnO: Er)_sapphire/silicon_ thin films are shifted at higher energy levels compared to pure ZnO, indicating that the behaviors of (ZnO: Er)_sapphire/silicon_ thin films are substrate dependent. XANES study of Zn *K*-edge/Zn *L*_3_-edge shows the transition of Zn 1*s* →4*p* / Zn 2*p* → Zn 4*s*3*d* in antibonding states of Zn; whereas Er *L*_3_-edge is the transition of 2*p*→5*d* states of Er upon x-ray absorption that demonstrates the local atomic structure around Er in the (ZnO: Er)_sapphire/silicon_ thin films. These transitions reveal the host-lattice in Zn-sites is substituted with Er^3+^-ions to preserve the symmetry with negligible distortion. The O *K*-edge shows the transition of O 2*p* - Zn 4*s* hybridized states through charge transfer the oxygen bands (Er-O-Zn) that reduced slightly atomic radius of Zn-O/Er-O/Zn-Zn found from extended x-ray absorption fine structure study, implies (ZnO: Er)_sapphire_ is higher ferromagnetic behaviour than (ZnO: Er)_silicon_. The defect emission bands also identified through Er-substitution indicated that the emissions changed the diamagnetic-ZnO into room temperature ferromagnetic DMSs (ZnO: Er)_sapphire/silicon_ thin film that could be useful for optoelectronic/magnetic applications.

## Introduction

Pure ZnO is a diamagnetic-semiconductor wide bandgap (∼3.37 eV) material with high exciton binding energy (≈60 meV), enables efficient excitonic emission at room temperature that makes it most suitable material for optoelectronic applications^1–3^. ZnO can be prepared in different forms, viz. single crystals, epitaxial films, along with various nanostructures, to meet different applications^4^. When ZnO is substituted with transition metals (TM: Fe, Co, Ni, etc.)^5–7^ and/or rare earth elements (RE: Er, Eu, Yb, etc.)^8–11^, then ZnO can act as a ‘diluted magnetic semiconductor’ (DMS) materials. It is noted that the 4*f-* RE-metals substituted ZnO have higher magnetic moments, compared with 3*d* TMs. The electrons could facilitate the ferromagnetic (FM) interaction among the RE ions as a result of the interaction between *f* electrons and host *s* electrons. Therefore, comprehending the electronic structure and magnetic characteristics of RE-substituted ZnO is crucial because of its prospective use in electronic/photo-electronic and spintronic devices. Especially, ZnO-substituted with RE erbium (ZnO: Er) can exhibit FM behaviors. The FM behaviours of ZnO: Er are often attributed to defects, like oxygen vacancies within the ZnO lattice, which can interact with the Er^+^-ions and facilitate spin alignment. Some studies report the observation of FM behaviors in ZnO: Er at room temperature. However, the exact magnetic mechanism and extent of this FM can depend on the synthesis methods, temperature, and Er^+^-concentration. In case of thin film, the magnetic behaviors are dependent not only on these parameters but also on the nature of the substrate used for the deposition of ZnO: Er thin films, as the substrate’s properties can affect the film’s structure, strain, defects, and ultimately, its magnetic properties. The substrate can also influence the magnetic anisotropy of the film, making it easier for the magnetization to align in a particular direction. In ZnO: Er thin films, the Er-dopant introduces magnetic moments that can interact with the surrounding ZnO matrix. The substrate can influence the interaction between Er-ions and the ZnO matrix, as whole affecting the magnetic properties of the film. The study of magnetic properties in ZnO: Er is an active research field for potential applications in electronic and spintronics due to its ability to manipulate both the charge and spin of electrons. The magnetic moment arises in Er-substituted ZnO from the localized unpaired 4*f* electrons of Er^+^-ions substituting Zn-sites in the lattice structure of ZnO. These Er^+^-ions are localized after substitution and can align with an applied magnetic field. However, the exact mechanism behind the ferromagnetism in Er-substituted ZnO is still under investigation, and achieving stable ferromagnetism at room temperature remains challenging. Among the various deposition techniques, sputtering is a potential technique, which can form thin film with strong adhesion layer^12^. The quality of thin films is affected by a number of sputtering parameters, including sputtering power, substrate temperature, chamber pressure, and sputtering time^13^. Because the nature of the substrate has a significant impact on the properties of thin films, selecting the appropriate substrate is crucial in many applications because the quality of the thin film nucleation/growth behavior depends on the substrate’s orientation, roughness, cleanliness, and temperature^12–18^. The thermal expansion coefficient mismatch between a ZnO thin film and its substrate significantly impacts the thin film’s quality^14^. Therefore, it affects the film’s structural, optical, electrical and magnetic properties.

In this present study, we have grown RE element Er-substituted polycrystalline-ZnO (ZnO: Er) thin films on two different substrates, viz. sapphire and silicon [(ZnO: Er)_sapphire/silicon_], using the DC sputtering technique. Sapphire and silicon substrates were chosen to see the crystal strain effect on deposited materials as there is a large lattice mismatch between ZnO and (ZnO: Er)_sapphire/silicon_. These choice of substrates are also used to study the grown of the thin films on a particular orientation. Therefore, the substrate effects of sapphire and silicon on the structural, electronic/atomic, and magnetic properties of RE element Er-substituted polycrystalline-ZnO [(ZnO: Er)_sapphire/silicon_], thin films have been studied. We have discussed several effects associated with the ZnO materials for the development of ZnO-based dilute magnetic semiconductors (DMSs) and their device-based applications. A comparative electronic/atomic/bonding-structure of (ZnO: Er)_sapphire/silicon_ thin films has been studied using X-ray absorption near edge structure (XANES) and X-ray photoelectron spectroscopy (XPS) measurements, whereas magnetic behaviors are studied using SQUID-measured magnetic hysteresis M-H loops. XANES spectroscopy is an ideal technique because it is an element-specific probe that is highly sensitive to the local structure around the absorbing crystalline and non-crystalline atoms^19,20^. In addition, crystalline surface morphology is studied using scanning electron microscopy (SEM) images. The evolution of local electronic structures is proposed, where the Er in (ZnO: Er)_sapphire/silicon_ thin film could behave as DMSs and could be used in electronic/spintronics applications.

## Results and discussion

**Fig. 1:**
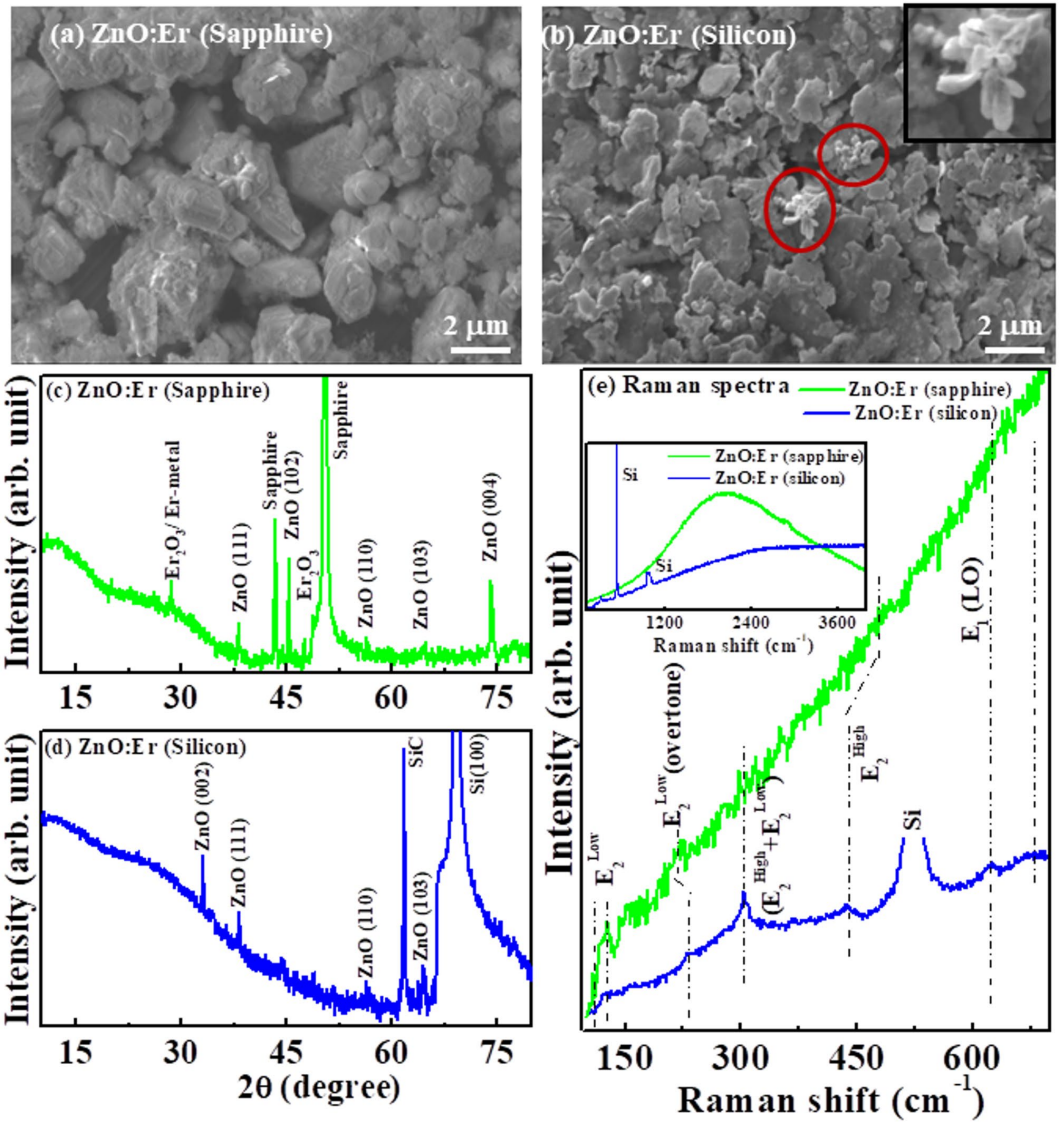
(**a**) Scanning electron microscopy (SEM) of ZnO: Er deposited on (**a**) Sapphire substrate and (**b**) Silicon substrate. XRD patterns of ZnO: Er (**c**) On sapphire and (**d**) On silicon. Raman spectra of ZnO: Er deposited on sapphire and silicon substrate.

Figure 1a,b shows the SEM micrograph of (ZnO: Er)_sapphire/silicon_ thin films. Differences in micrograph size/textures are due to the different substrate’s mismatch in lattice, stress, Er-content present (at%) in the ZnO matrix, and the thermal expansion coefficient between the ZnO: Er thin film and the substrates (sapphire/silicon). From the inset Fig. 1b, a branch of leaf-shaped cone arm structure with non-uniform diameter along its axes is observed in the (ZnO: Er)_silicon_ thin film matrix, while no such structure is observed in (ZnO: Er)_sapphire_. Zhuo et al.^21^. also observed similar four legs regular-shaped hexagonal cone arm and defined as the tetrahedral structure in nanostructures-ZnO materials. The XRD spectra of (ZnO: Er)_sapphire/silicon_ is shown in Fig. 1c,d. Different (ZnO: Er)_sapphire/silicon_ diffraction peaks and their orientations are identified/indicated in the XRD spectra^22–26^, as shown in Fig. 1c,d. It is observed that both the films (ZnO: Er)_sapphire/silicon_ are partially oriented epitaxial polycrystalline hexagonal wurtzite structure, exhibiting (002), (111), (102), (110), (103), (004) orientations. Moreover, the XRD patterns of (ZnO: Er)_sapphire_ is grown along (111) plane; whereas (ZnO: Er)_silicon_ is grown along (002) and (111) planes. In addition, some other additional minor peaks of ZnO and the substrate (sapphire/silicon)-peaks are also observed in the respective (ZnO: Er)_sapphire/silicon_ thin films. The (ZnO: Er)_sapphire_ thin films provide better crystalline quality and surface morphology compared to (ZnO: Er)_silicon_ thin films. The silicon-substrate is a cost-effective and readily available option, especially for certain applications where UV performance is not the primary concern; whereas sapphire has a wurtzite structure, which aligns well with the wurtzite structure of ZnO film that facilitates better growth with smoother surfaces, leading better crystalline quality with reduced threading dislocations, improved crystalline orientation and larger grain sizes compared to those grown on silicon. In this context the lattice constants, crystallite size (from Scherrer’s formula) and the strains are obtained from XRD analysis and tabulated in Table 1 to support crystalline quality of the (ZnO: Er)_sapphire/silicon_ thin films. It is clearly observed that the micro-strain is induced in (ZnO: Er)_Sapphire_ thin film is approximately two times larger than (ZnO: Er)_Silicon_ thin film which are the responsible for growing most preferentially orientation along (111) plane in case of sapphire substrate and (002), (111) plane in Si-substrate. From the obtained **c/a** values (see Table 1), it is observed that the ZnO structure is distorted in (ZnO: Er)_sapphire_ thin film compared to (ZnO: Er)_silicon_ thin film. It is well known that the oxide of Er i.e. Er_2_O_3_ exhibit anti-ferromagnetic at low temperature (~ 3.3 K) and paramagnetic at room temperature or above of 3.3 K^27^, as a result the magnetic contribution from Er_2_O_3_ in this present work is ruled out. Again, the Er-cluster or Er-metal exhibits both ferromagnetic as well as paramagnetic at low temperature^28^. As, we have not observed Er-clusters or Er-atomic cluster, so it is also ruled out in the present study. The Raman spectra of (ZnO: Er)_sapphire/silicon_ thin films are shown in Fig. 1e. The spectral features shows that the (ZnO: Er)_sapphire/silicon_ thin films are highly photoluminescence (PL) behaviours as inset Fig. 1e, while (ZnO: Er)_sapphire_ is higher PL than (ZnO: Er)_silicon_ indicating the formation of highly defective (ZnO: Er)_sapphire_ thin film. In a (ZnO: Er)_sapphire_ thin film, Raman mode peaks are difficult to observe due to the dominance of PL intensity over the Raman scattering signal. The presence of this high PL behaviour, particularly in (ZnO: Er)_sapphire_ thin film is due to formation higher defective thin films compare to (ZnO: Er)_silicon_ and responsible for the higher ferromagnetic behaviors^29–34^. All the observed spectroscopic Raman peaks are identified in Fig. 1e can be assigned to a wurzite ZnO structure^35–37^. However, in the Raman spectra the peak at 99 cm^−1^ is associated to the E_2_ (low) dominated by the vibrations of the heavy Zn-sub-lattice and the band at around 438 cm^−1^ attributed to the E_2_ (high) mode, mostly involves the oxygen atoms of (ZnO: Er)_sapphire/silicon_ thin film^35–37^. A significant shift 483 cm^−1^ for the (ZnO: Er)_sapphire_ is observed due to the formation of different phase of ZnO: Er films. The peak 620 cm^−1^ is ascribed as the combination of optical-acoustic mode^38^. The peak at 200 cm^−1^ is the overtone of the E_2_ (low) and the peak at 336 cm^−1^ is due to the combination E_2_ (high) and E_2_ (low) modes^37,38^. The E_2_ (high) mode centered at 438 cm^−1^ has a stronger intensity and narrower line-width in (ZnO: Er)_silicon_ film, which indicates that the grown of ZnO: Er is a hexagonal wurtzite structure^37,38^.

**Table 1:** The values of cell parameters from XRD, crystallite size, strain for ZnO:Er deposited on sapphire and silicon substrate.

Sample specification	Cell parameters (Å)	***c***/***a***	Unit cell volume (Å^3^)	Crystallite size (t= 0.89λ/ βcosθ) (nm)	Strain (ε=βcosθ/4sinθ)
ZnO:Er on sapphire substrate	a = 3.271 ±0.0002 c = 5.132 ±0.0002	1.56	47.57±0.006	105 nm	1.06 × 10^−3^
ZnO:Er on silicon substrate	a = 3.270 ±0.001 c = 5.239 ±0.0004	1.60	48.55±0.005	93 nm	5.4 × 10^−4^

**Fig. 2:**
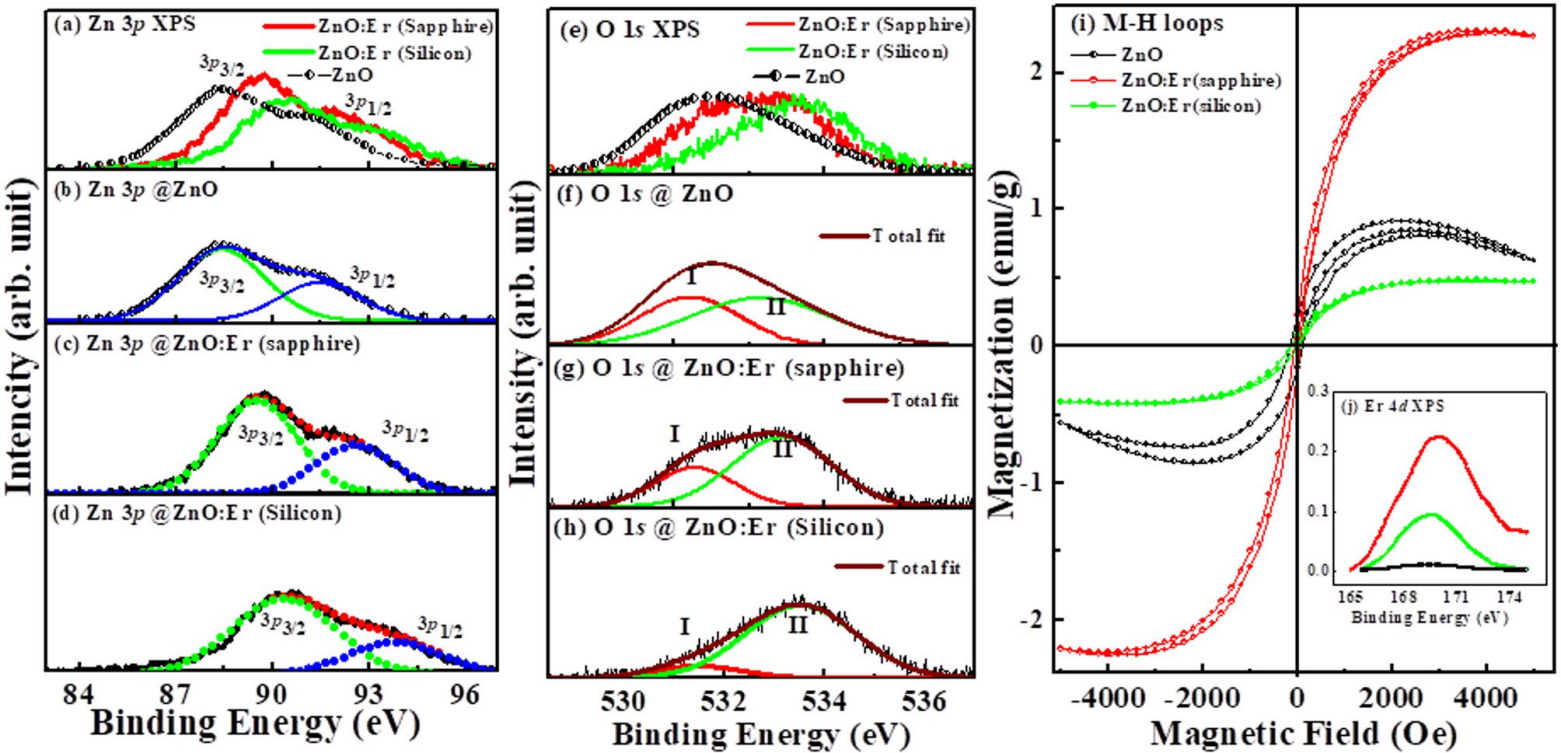
(**a**) X-ray photoelectron spectroscopy (XPS) of (**a**) Zn 3*p* of ZnO, ZnO: Er; Zn 3*p* decomposed into Gaussian features (**b**) pure-ZnO, (c) ZnO: Er (Sapphire), (**d**) ZnO: Er (silicon). XPS of (**e**) O 1*s* of ZnO, ZnO: Er; O 1*s* decomposed into Gaussian features (**f**) pure-ZnO, (**g**) ZnO: Er (Sapphire), (**h**) ZnO: Er (silicon). (**i**) Magnetic M-H hysteresis loops of ZnO, ZnO: Er (Sapphire), and ZnO: Er (silicon). (**j**) Inset XPS of Er 4*d*.

XPS has been used to analyse the chemical composition (Zn, O, Er), quantification (at%), electronic structure and their defects. The quantificational analysis of XPS spectra shows ≈ 2.5 at% and ≈ 1.0 at% Er is substituted in (ZnO: Er)_sapphire_ and (ZnO: Er)_silicon_ thin films, respectively, although the deposition parameters/conditions remained unchanged during the deposition of (ZnO: Er)_silicon/sapphire_ thin films. It is also observed that the Er-content in the film structure is not only different at%, but their peak positions in Zn 3*p* and O 1*s* XPS spectra of (ZnO: Er)_sapphire/silicon_ as shown in Fig. 2a-d, e-h) respectively, are shifted at higher energy levels compared to pure ZnO, indicating that the deposition of ZnO: Er thin films are substrate-dependent. The corresponding Zn 3*p* and O 1*s* XPS spectra and their convolutions are shown in Fig. 2a–d and e–h, respectively. The Zn 3*p* spectrum for pure ZnO consists of two strong peaks at ≈88.4 eV and ≈91.5 eV, correspond to Zn 3*p*_3/2_ and Zn 3*p*_1/2_ states, respectively. The difference between the Zn 3*p*_3/2_ and Zn 3*p*_1/2_ binding energies found to be ≈ 3.1 eV, which closely matches with the value reported earlier^39,40^. Oxygen in the ZnO matrix is coupled to Zn^+^-ions in the + 2-oxidation state, as indicated by the locations of both peaks and the difference in their binding energies^39,40^. The Zn 3*p* spectral features of (ZnO: Er)_sapphire/silicon_ thin films closely resemble each other with red-shift ≈ 0.8 eV [3*p*_3/2_: ZnO (pure), 88.4 eV →(ZnO: Er)_sapphire_/(ZnO: Er)_silicon_, 89.5 eV/90.3 eV; 3*p*_1/2_: ZnO (pure), 91.5 eV →(ZnO: Er)_sapphire_/(ZnO: Er)_silicon_, 92.5 eV/93.8 eV] and increase their peak widths [ZnO→(ZnO: Er)_sapphire_→(ZnO: Er)_silicon_] with variations of their intensities. This suggests that the introduction of Er influences the electronic states of Zn due to the existence of Zn interstitials/vacancies. The O 1*s* peak at 531.4 (± 0.1) eV of pure ZnO is believed to be due to the wurtzite structure of hexagonally arranged Zn^2+^ ions in the metal oxide as confirmed from XRD results. Different components, such as oxygen vacancies and absorbed oxygen/hydroxyl groups^41–43^, could be the reason for the other peak at higher binding energy, 533.0 (±0.5) eV. Based on existing literature, it is inferred that the peak is associated with oxygen vacancies in the system^42,43^. The incorporation of Er-ions into the ZnO lattice results in a heightened intensity of the peak at elevated binding energy, signifying an increase in oxygen vacancies. This increase in oxygen vacancies is, in turn, responsible for the improved saturation magnetization associated with Er. This implies higher room temperature ferromagnetism in (ZnO: Er)_sapphire/silicon_ thin films compared to diamagnetic ZnO. The room temperature (RT) magnetic behavioral M-H hysteresis loops of ZnO (ZnO: Er) are shown in Fig. 2i and the Er 4*d* XPS spectrum of (ZnO: Er)_sapphire/silicon_ thin films are shown in Fig. 2j inset in Fig. 2i. In the Er 4*d* XPS spectrum, the binding energy of ≈ 169.4 eV is attributed to the 4*d* levels of Er^3+^ ions are in a trivalent (Er^3+^) state in (ZnO: Er)_sapphire/silicon_ thin films *via* an interaction with an unfilled shell. Thus, we can deduce that the RT-FM is a fundamental characteristic of the materials involved by Er³⁺ ions and may be in a high-spin configuration (4*f*^11^6*s*^0^). While Er³⁺ ions contribute to magnetism, the mechanism is complex and often involves other factors like substitutions, carrier mediation and unpaired electron, rather than simply a high-spin configuration. In the RT magnetic M-H hysteresis loops, the contribution of the diamagnetic signals of substrates was adequately corrected. It is observed that the magnetic moment (M_S_ =2.3 emu/g) and coercivity (H_C_ =63 Oe) of (ZnO: Er)_sapphire_ thin film is higher than (ZnO: Er)_silicon_ thin film (M_S_ =0.5 emu/g, H_C_ =15 Oe) that confirms the magnetism is also substrate dependent. This observation is consistent with Er content (at %) obtained from XPS elemental/quantitative analysis, which indicates that the Er at% is substrate dependent. The (ZnO: Er)_sapphire_ has a higher Er (~ 2.5 at%), and the magnetization is also higher than the films deposited on a silicon substrate with an Er concentration of ~ 1.0 at%. In the context of Er-substitution, the predominant defects in ZnO are probably oxygen vacancies (V_0_) and zinc vacancies (V_Zn_). Specifically, V_0_ is expected to be more prevalent in Zn-rich environments due to its lower formation energy compared to zinc interstitials, while V_Zn_ is likely to be more prominent in oxygen-rich conditions. Thus, it is plausible to infer that there may be a significant presence of zinc and oxygen vacancies in (ZnO: Er)_sapphire/silicon_ thin films. Our experimental findings may align with the bound-magnetic-polaron (BMP) model suggested by Coey et al.^44^, as the presence of both zinc and oxygen vacancies promotes the development of ferromagnetism in diluted magnetic semiconductors (DMSs)^45,46^. It is noted that the magnetic contribution is not due to Er-oxide and/or Er-metal. As Er has more affinity towards oxygen, so oxygen deficiency occurred in parent ZnO. Higher Er-content leads to higher oxygen deficiency and forms oxygen vacancy clusters which are responsible for observation of ferro-magnetic behavior. This is the reason why we have given much emphasis on vacancy mediated ferromagnetism. According to the BMP model, magnetic exchange can be mediated by shallow donors (Zn_i_) electrons that form bound magnetic polarons, and the presence of dopant, i.e., the magnetic exchange interaction between Zn or O vacancies of Er³⁺ ions occupying the same space, is aligned with Er spins^3+^, forming BMPs. With the reduction of V_0_ and V_Zn_ by Er-substitution in ZnO, neighboring Er-ions coupled *via* a V_0_ and V_Zn_ (ferromagnetic exchange) are connected by a zinc bond (no exchange interaction) or oxygen bond (super-exchange interaction), which are responsible for the alteration of M_S_ values. It is noted that the exchange interaction has not arisen due to Er. Due to high oxidation state of Er, the oxygen deficiency has been observed in the ZnO structural matrix. The exchange interaction is Er coupled and/or mediated which is responsible for the observation of ferromagnetism. The substrate can also influence the formation and characteristics of these defects, thus affecting the BMP formation and the overall magnetic behavior. In the case of (ZnO: Er)_silicon_, the ferromagnetic behavior is weakened, possibly due to Er ions^3+^ near the surface sites with their ionic radii mismatch^47^.

**Fig. 3:**
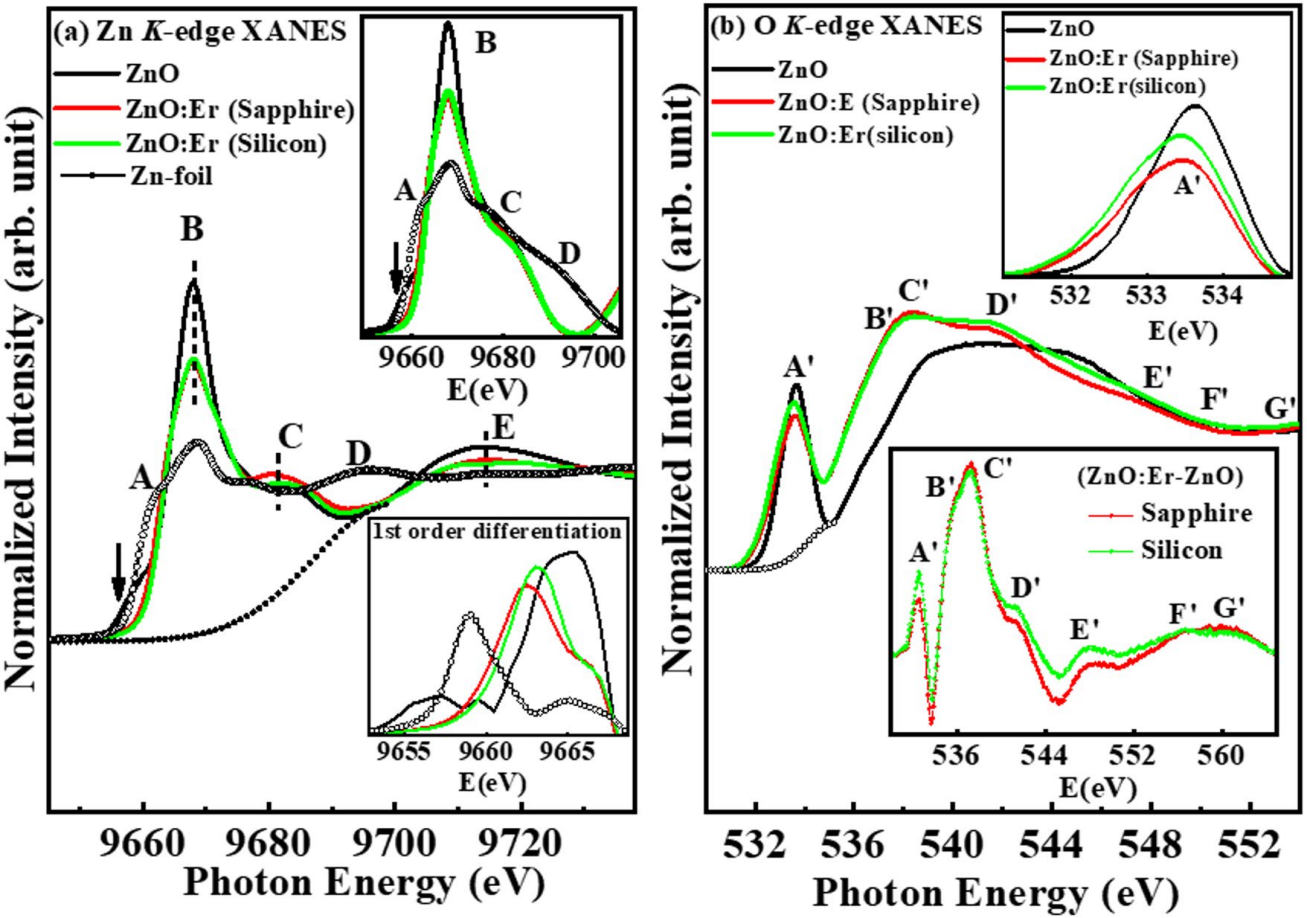
(**a**) Zn *K*-edge X-ray absorption near edge structure (XANES) spectra of ZnO and ZnO: Er deposited on sapphire/silicon substrate. The lower inset displays the 1 st order differentiation of the Zn *K*-edge XANES spectra and upper inset display a magnified view of the pre-edge features after the background has been subtracted using a best-fitted Gaussian line. (**b**) O *K*-edge XANES spectra of ZnO and ZnO: Er deposited on sapphire/silicon substrate. The upper inset displays a magnified view of the pre-edge features after the background has been subtracted using a best-fitted Gaussian line, whereas the lower inset plots differences of O *K*-edge between ZnO: Er and ZnO (ZnO: Er-ZnO).

To investigate the electronic/atomic structures and gain insight into their microstructure, we have used the Zn and O *K*-edges, Zn and Er *L*_3_-edge XANES spectra, along with Zn *K*-edge and Er *L*_3_-edge EXAFS spectra of ZnO (ZnO: Er)_sapphire/silicon_ thin films. The Zn *K*- and O *K*-edges XANES spectra of ZnO (ZnO: Er)_sapphire/silicon_ thin films are shown in Fig. 3a,b. As a reference, the Zn *K*-edge XANES spectrum of Zn-foil (Zn^0^) is also shown in Fig. 3a. To identify the proper absorption edge, first-order differentiation of Zn *K*-edge XANES spectra is shown in the lower inset of Fig. 3a. The transition of Zn 1*s* →Zn 4*p* dominates the Zn *K*-edge absorption spectra, which show the excited 1*s* core electrons of the zinc atoms. The Zn 1*s*→ 4*p* transitions are the distinctive peaks that are seen on the leading absorption edge. ༴he fully populated 3*d* levels of metal Zn-atoms (Zn^0^) are pushed below the Fermi level. Nevertheless, the zinc atoms’ oxides produce empty states in the 3*d* levels, Zn^2+^, because their electrons are required for bonding. Therefore, one might assume that an electron excited from the core levels would have a *d*-level state. However, quantum selection criteria, which prohibit *s* electrons from moving in *d* shells (i.e., Δl = ± 1) prevent the 1*s* → 3*d* transition. The Zn *K*-edge absorption edge of Zn-foil (Zn^0^) and pure-ZnO are observed at ≈ 9659.1 eV and ≈ 9665.5 eV respectively, along with a weak pre-edge at ≈9657.0 eV (marked with a black line), which are the hybridization of O-2*p* and Zn^2+^ 3*d* orbitals^48^. The ZnO exhibits molecular bands characterized by narrower 4*p* levels, with the closest neighbouring atoms being oxygen. The outer-shell electrons of the oxygen atoms are confined to the 2*p* levels, while the unoccupied levels on the zinc atoms correspond to the 4*p* levels. Quantum selection rules arise due to an interaction between the *p*-levels, which results in the narrowing of the 4*p* levels in ZnO materials (i.e. Zn^2+^). The absorption edges of Er-substituted ZnO, (ZnO: Er)_sapphire/silicon_ are observed at ≈ 9663.0 eV/9662.5 eV, in between Zn-foil and pure ZnO as shown in the upper inset of Fig. 3a. The edge shifts from ≈ 9665.5 eV (ZnO) → ≈9663.0 eV/9662.5 eV (ZnO: Er)_silicon/sapphire_ indicate the oxidation states of ZnO: Er (between Zn^2+^ and Zn^0^) are decreased on (ZnO: Er)_silicon/sapphire_ thin films compared to pure ZnO. The oxidation states of (ZnO: Er)_sapphire_ thin film is nearer to Zn-foil (Zn^0^) than (ZnO: Er)_silicon_, due to the edge shift being ≈ 0.5 eV lower than (ZnO: Er)_silicon_. It is also noticed that the pre-edge of Er-substituted ZnO is merged with the absorption edge in both (ZnO: Er)_sapphire/silicon_ films. Similar edge shifts are also observed in O *K*-edge XANES spectra, illustrate in the upper inset Fig. 3b, provide a magnified perspectives of the pre-edge characteristics following the subtraction of the background as lower inset Fig. 3b, which has been accomplished using a best-fitted Gaussian curve as denoted by the dotted line. This variation in the general line shapes, the absorption intensity, and the positions of the peaks of the Zn and O *K*-edge XANES spectra, as shown in Fig. 3a, b of the pure-ZnO and (ZnO: Er)_sapphire/silicon_, has been regarded as evidence of substrate-dependent in O/Zn-*p* states. According to the dipole-transition selection rule, features A, B, C, and D, as shown in Fig. 3a, can be attributed to the Zn 4*p-*derived state. In Zn *K*-edge, the main feature at ≈ 9668.0 eV (marked as B) is attributed to the Zn-atoms in tetrahedral site symmetry of ZnO^49^. The upper inset of Fig. 3a shows a comparison of Zn *K*-edge spectra of ZnO and (ZnO: Er)_sapphire/silicon_ thin films. A detailed view of the near-edge characteristics following the subtraction of the background, which has been accomplished using a best-fitted Gaussian curve as represented by the dotted line. No pre-edge feature is observed in (ZnO: Er)_sapphire/silicon_, but peak shifts occur on the (ZnO: Er)_sapphire/silicon_ thin films. The peaks at ≈ 9683/9712 eV (marked as C/D) of ZnO/(ZnO: Er)_silicon_ films because of the diverse scattering effects of the photoelectrons in the neighboring atoms environment that shifted to ≈ 9681/9714 eV for the (ZnO: Er)_sapphire_ films. We have obtained the intensities by integrating the area within the range 9648.5–9595.5 eV for ZnO, 9653.0–9697.0 eV for (ZnO: Er)_sapphire/silicon_ thin film and found that the intensity of ZnO: Er is reduces from ZnO (29 unit) → ZnO: Er (sapphire/silicon: 25.5/25.3 unit). Again, the intensity of (ZnO: Er)_sapphire_ films is higher than the films (ZnO: Er)_silicon_, which implies an increase of the Zn 4*p*-orbital occupation or the positive effective charge on the Zn-ions that supports the formation of higher ferromagnetic behavior in ZnO: Er thin films. Figure 3b shows O *K*-edge XANES spectra of ZnO/(ZnO: Er)_sapphire/silicon_. In accordance with the dipole-transition selection rule, the distinct peaks observed in the energy range 531–565 eV originate from O 1*s*-derived states transitioning to 2*p*_σ_-derived (along the bilayer) and O 2*p*_π_-derived (along the ***c*** axis) states^50–52^. The contributions in the absorption edge mainly arise from O 2*p* - Zn 4*s* hybridized states^53^. The strong features A is due to the asymmetry of the wurtzite structure. The absorption edges are shifted to lower energy, when Er is substituted with ZnO, which can be seen in an enlarged depiction of the pre-edge (feature-A′) after the background has been eliminated, utilizing a best-fitted Gaussian curve as indicated by the dotted line as shown in the upper inset of Fig. 3b. The fact that the intensity of (ZnO: Er)_sapphire/silicon_ thin films (feature-A′) was found to be lower than pure ZnO (upper inset Fig. 3b) indicating the total number of unoccupied O 2*p*-derived states is decreased, which can be seen as the transfer of electrons from Er → O 2*p* states due to O 2*p*-Er 5*d* hybridizations in (ZnO: Er)_sapphire/silicon_ thin films^50,51^. The lower inset Fig. 3b shows the differences in the O *K*-edge near edge features of [(ZnO: Er)_sapphire/silicon_ - (ZnO)] thin films. The overall spectral intensity of (ZnO: Er)_sapphire/silicon_ has been reduced and the near-edge feature has been broadened in comparison to those of ZnO, indicating a reduction in the number of unoccupied O 2*p* states because of the Er-diffusion and heightened negative effective charge of the O ion. The rise in the effective charge of the O ions is explained by the observation that Pauling’s electronegativity of Er (1.24) is considerably less than that of Zn (1.65)^54^. The enhancement of these characteristic features (A′, B′, C′, D′, E′, F′, G′) signifies the elevated local density of states (DOS) that originate from the defects and/or dangling bonds present in ZnO/(ZnO: Er)_sapphire/silicon_ thin films^55^. Again, intensities of the features B′, C′, G′ is higher in (ZnO: Er)_sapphire_ thin films compared to (ZnO: Er)_silicon_ films; whereas the features A′, D′, E′, F′ of the (ZnO: Er)_silicon_ films is higher than (ZnO: Er)_sapphire_ films, indicating the population of defects and/or vacancies are substrate dependent. The overall intensities of (ZnO: Er)_sapphire_ film is lower than (ZnO: Er)_silicon_ suggesting a rise in the occupancy of the O 2*p* orbitals and an increase in the negative effective charge of the O-ion that supports the higher ferromagnetism in the (ZnO: Er)_sapphire_ films.

**Fig. 4:**
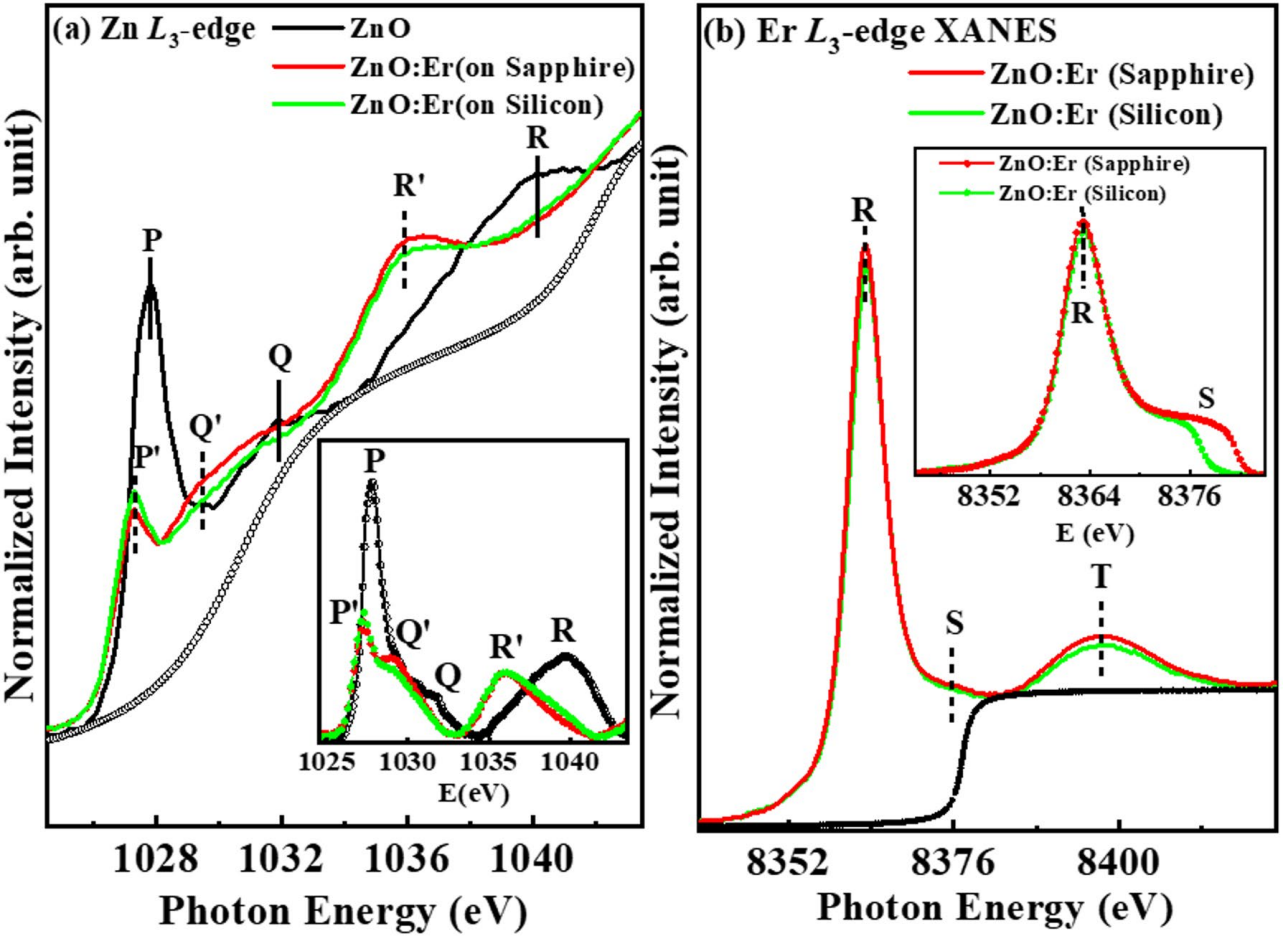
(**a**) Zn *L*_*3*_-edge and (**b**) Er *L*_*3*_-edge X-ray absorption near edge structure (XANES) spectra of ZnO and ZnO: Er deposited on sapphire/silicon substrate. Inset (**a**, **b**) display a magnified view of the Zn *L*_*3*_ and Er *L*_*3*_ pre-edge features after the background has been subtracted using a best-fitted Gaussian line.

Figure 4a,b shows Zn *L*_3_-edge and Er *L*_3_-edge XANES spectra of ZnO and (ZnO: Er)_sapphire/silicon_ thin films. XANES measurements examine the Zn *L*-edge, providing knowledge of the unoccupied Zn *d* and Zn *s*-states. The Zn *L*_3_ and *L*_2_ regions, correspond to the Zn 2*p* → Zn 4*s* and Zn 2*p* → Zn 3*d*anti-bonding states, respectively^56^. The spectral characteristics observed in the *L*_2_ region indicate the electron transition from Zn 2*p* state to Zn 3*d* state. No pre-edge feature is observed in ZnO/(ZnO: Er)_sapphire/silicon_ implying no Zn 4*s* band transition occurs. The Zn *L*_3_-edge absorption peaks arise due to the transition of the 2*p* core electron to the unoccupied 4*s*/4*d* levels as final states. In accordance with the dipole-transition selection rule, Zn *L*_3_-edge XANES investigates the unoccupied states derived from Zn *s*- and *d*-orbitals. The Zn-3*d* orbital is completely filled, making the lowest unoccupied orbital of the Zn-ion is Zn-4*s*, succeeded by Zn-4*p* and Zn-4*d*^57^. The degenerate states 2*p*_3/2_ and 2*p*_1/2_ emerge due to spin-orbit coupling, leading to multiplets centered at ≈1027.8 eV and ≈1032.0 eV. The crystal field (octahedral) raises the degeneracy of 2*p*_3/2_ and 2*p*_1/2_ levels, creating of *t*_2g_ and *e*_g_ sub-band symmetries^58^. The fine structure multiplets arise from two effects: (i) interaction between the 3*d* (electron) and 2*p* core hole, and (ii) crystal field created by neighboring ions at a Zn^2+^ site^59^. The inset at the bottom of Fig. 4a is a magnified view of the near-edge features after subtracting the background using a best-fitted Gaussian curve indicated by the dotted line. It is found that the absorption features A/B(wide range) are observed at ≈1027.8/1031.9 eV in ZnO, which is shifted towards lower energy level (A′/B′) at ≈1027.3/1028.9 eV for the (ZnO: Er)_sapphire_ film and ≈1027.3/1029.0 eV in (ZnO: Er)_silicon_ film, respectively. The overall peak width and intensities of (ZnO: Er)_sapphire/silicon_ thin films are decreased compared to pure-ZnO, indicating that the quantity of unoccupied Zn 4*sd* states in proximity to the conduction-band minimum is lowered as the Er is substituted with ZnO. We have integrated the Zn *L*_3_-edge region and found that the intensity of pure ZnO ≈ 0.77 (a.u.) is higher relative to (ZnO: Er)_sapphire/silicon_ ≈0.49/0.47 (a.u.). The intensity of (ZnO: Er)_sapphire_ films higher than the (ZnO: Er)_silicon_, indicating that the unoccupied Zn 4*sd* states are higher in (ZnO: Er)_sapphire_ than (ZnO: Er)_silicon_. The augmentation of the defining features denotes the heightened local density of states (DOS) that originate from defects or dangling bonds within various ZnO nanostructures^51^. The (ZnO: Er)_sapphire_ thin film shows higher absorption intensity concerning the (ZnO: Er)_silicon_ thin film, signifying a greater prevalence of defects and/or vacancies at O sites in (ZnO: Er)_sapphire_ thin film. As a result, the ferromagnetic nature is stronger in the (ZnO: Er)_sapphire_ thin film than (ZnO: Er)_silicon_. To investigate the local electronic structure of Er in ZnO, we used the XANES spectrum of Er-substituted ZnO at Er *L*_3_-edge. The variation of white line intensity of (ZnO: Er)_sapphire/silicon_ thin films is at ≈8363.3 eV and ≈8398.0 eV respectively, as shown in Fig. 4b. The peaks at ≈8363.3 eV (R) and ≈8398.0 eV (T) arise due to the 2*p*−5*d* transition of Er upon x-ray absorption^60–66^. The magnitude of the white line peak signifies the occupancy of 5*d* valence electronic states, which is contingent upon the symmetry of the absorbing atom. A high-order symmetry, such as that of the ErO_6_ octahedron (O_h_ point group), results in a concentrated density of states (DOS) of the 5*d* states, attributed to the equivalent coordination of six O atoms around Er. This concentrated DOS leads to a significant pre-edge peak; nevertheless, the peak height of the (ZnO: Er)_silicon_ thin film is reduced as a result symmetry degradation^59^. The observed decrease in intensity to a reduced DOS concentration, which can be ascribed to the less symmetric geometry in the coordination^53,58^. Thus, the symmetry around Er increases for the (ZnO: Er)_sapphire_ thin film as shown in the inset lower of Fig. 4b, implying the higher DOS. This higher DOS (2*p*−5*d*) of (ZnO: Er)_sapphire_ thin film implies a higher ferromagnetic nature compared to the (ZnO: Er)_silicon_ film.

**Fig. 5:**
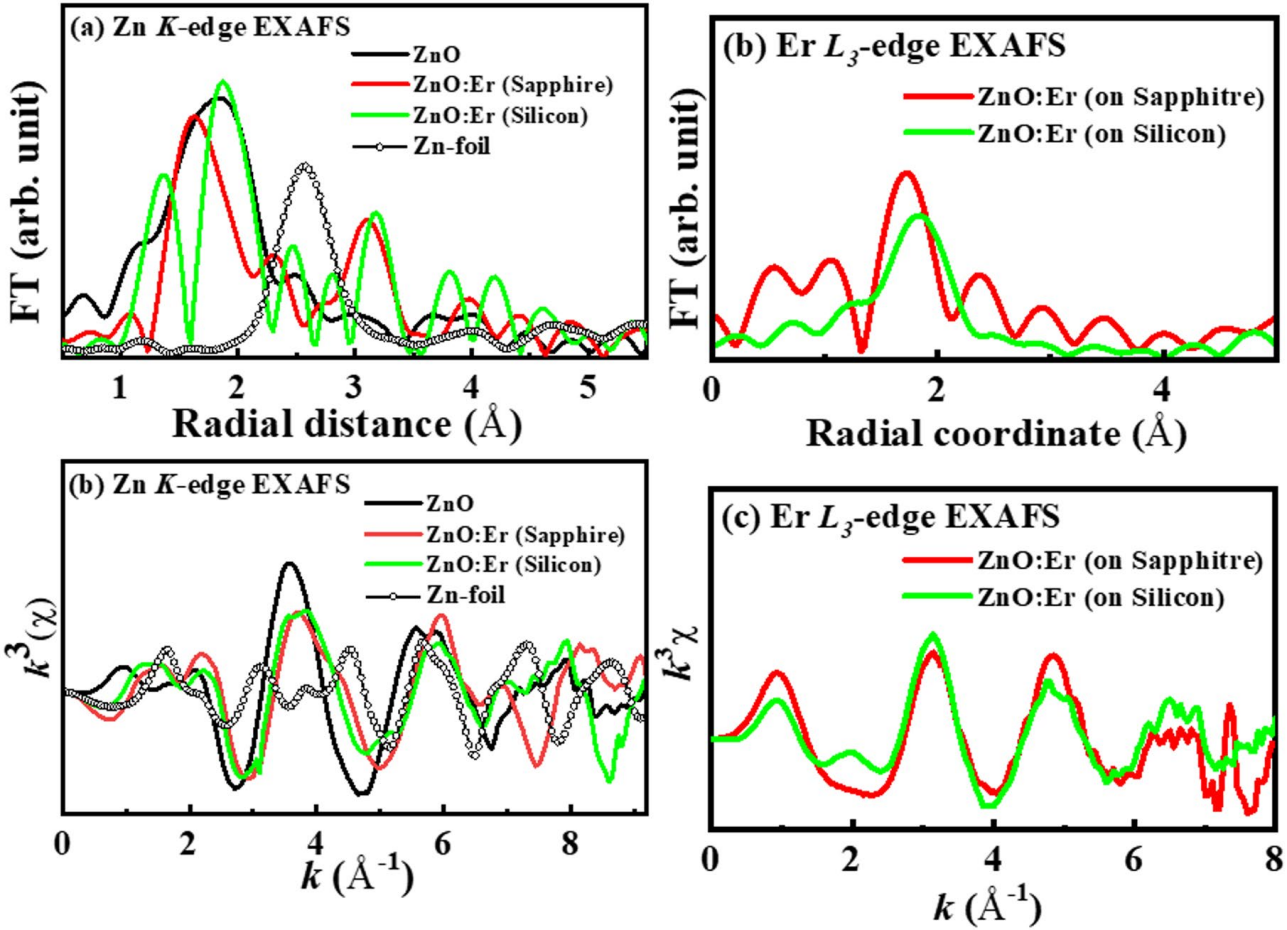
Magnitude of Fourier transform (FT) of EXAFS *k*^3^χ data at the (**a**) Zn *K*-edge and (**b**) Er *L*_3_-edge and (**c**, **d**) corresponding EXAFS data within the range k = 0 to 8 Å, weighted by ZnO and ZnO: Er deposited on sapphire/silicon substrate along with Zn-Foil.

The best tool for further study of the local structure around Zn atoms in Er-substituted ZnO is extended x-ray absorption fine structure (EXAFS) spectroscopy. The oscillations observed in EXAFS arise from the scattering and interference of photoelectron waves from source atoms with elevated kinetic energies. Due to their kinetic energy, photoelectrons can move between adjacent atoms. The outer shell electrons of these neighboring atoms exert a coulombic force on the incoming photoelectrons, which then follow a path characterized as either single or multiple scattering. The fluctuation EXAFS spectra can be extracted by using the ATHENA and ARTEMIS programs.67 In this case, Fourier transform of *k*^3^χ was performed, where *k* is the photoelectron wave vector, defined as *k* = (4*π* *m* (*E* − *E*_0_)/h)^1*/*2^, where *m* is the electron mass, *E* is the photon energy, *E*_0_ is the energy threshold of the absorption edge, and *h* is the Planck constant. This Fourier transform gives the radial structural function (RSF) around Er i.e., the atomic distribution. This involves the reduction of data from the absorption spectra and the use of Fourier transform to obtain the χ(R) versus R plots, the development of theoretical EXAFS spectra starting from a hypothesized crystallographic structure, and finally the fitting of the experimental χ(R) versus R data with the theoretical data using the ARTEMIS programs^67^. The fitting parameters consist of the coordination number (N), bond distances, and disorder (Debye-Waller) factors (σ^2^), which indicate the mean square fluctuations in the distances. To analyze the local structure around Zn and Er in detail, χ(R) versus R plots generated for ZnO and (ZnO: Er)_sapphire/silicon_ thin films from the µ(E) versus E spectra through the Fourier transform of the χ (k) versus *k* plots with appropriate *k*(*k*^3^) weighting are presented in Fig. 5, measured at the Zn *K*-edge and Er *L*_3_-edge. Figures 5a,b illustrate the amplitude of the Fourier transform (FT) obtained from the EXAFS *k*^3^χ data for ZnO and (ZnO: Er)_sapphire/silicon_ films, along with Zn-Foil as reference, and their respective oscillations at the Zn *K*-edge. The peak observed in Zn-foil at ≈2.58 Å is described as a Zn-Zn bond, and the peak at ≈1.84 Å observed in pure ZnO is defined as Zn-O coordination bond. It shows that the line shapes and radial distribution of the FT spectra at Zn *K*-edge of the Er-substituted ZnO are not identical to ZnO, but with a slight change in their intensities and peak positions. The Zn-O bond lengths of ZnO: Er films are reduced to ≈1.63 Å/1.87Å (sapphire/silicon-substrate) compared to pure ZnO films (≈1.84 Å), but the Zn-Zn bond length increased to 3.09 Å/3.16 Å [(ZnO: Er)_sapphire_/(ZnO: Er)_silicon_] compared to Zn-foil (≈2.58 Å)^48^. In addition, one extra peak at ≈1.35 Å is observed in (ZnO: Er)_silicon_films, equivalent to the weak peak at ≈1.15 Å observed in pure ZnO films and also defined as the Zn-O bond^50^. These results suggest that the Er-atoms take the place of the host Zn ions in the core of ZnO: Er; the local atomic configuration at the Zn sites is markedly distorted, owing to the substantial difference in ionic radii of Er^3+^ (0.89 Å) and Zn^2+^ (0.74 Å). It is considered that the Er atoms substituted at the Zn sites on the surface layer and formed dangling bonds/defects with a high degree of disorder. The EXAFS *k*^3^χ data as shown in Fig. 5b indicates the spectral features of (ZnO: Er)_silicon_ film is identical with pure ZnO but non-identical with (ZnO: Er)_sapphire_ film indicates along with a slight phase shift in scattering data beyond *k* = 6 Å^−1^. This indicates a minor disturbance on the formations at closer distance to the source Zn atoms in (ZnO: Er)_sapphire/silicon_ thin films. An observable shift at the Zn-O bond peak position points out a disturbance in the atomic locations. It is highlighted a displacement of defects in (ZnO: Er)_sapphire/silicon_ compared to the host elements of the ZnO materials in their wurtzite crystal structure with Er-substitution. These disorders and/or defects are higher in (ZnO: Er)_sapphire_ thin film, implying higher ferromagnetic behaviors. Figure 5c shows the radial structure function of (ZnO: Er)_sapphire/silicon_ and Fig. 5d is the *k*^3^-weighted EXAFS spectra of (ZnO: Er)_sapphire/silicon_. In EXAFS, the peak between 1.4 Å and 2.2 Å is the contribution of oxygen atoms in the ZnO: Er system^53^. The nearest-neighbor atom from Er is found at ≈ 1.7 Å/1.84 Å [(ZnO: Er)_sapphire_/(ZnO: Er)_silicon_] consistent with the observations reported by other groups^61^, where they found that the first neighbor atom should be O. In addition, a shoulder peak at ∼2.4 Å was observed near the first nearest-neighbor peak in (ZnO: Er)_sapphire_ films, suggesting the structural distortion of pseudo-octahedral^55,60^, implying the formation of higher ferromagnetic nature. The Fourier transformed (FT)χ(k) with *k* weight as shown in Fig. 5d, gives a pseudo-radial structure function around Er^59^. In this, a slight phase shift in scattering data beyond k = 6 Å−1 indicates a slight disturbance of the atoms closer to the source Zn atoms in (ZnO: Er)_sapphire/silicon_ thin film.

In conclusion, the DC sputtering method successfully synthesized the pure ZnO and (ZnO: Er)_sapphire/silicon_ thin films. ZnO was crystallized in the hexagonal wurtzite structure of ZnO without any impurity phases. The most common defects in ZnO/(ZnO: Er)_sapphire/silicon_ thin films are V_O_/V_Zn_ vacancies, which are confirmed from the transitions of Zn 1*s*→Zn 4*p*, Zn 2*p* → Zn 4*s*, Zn 2*p* → Zn 3*d*, and O 1*s* →2*p* in XANES study and peak shifts of Zn 2*p*/O 1*s* at higher binding energy level in XPS study. In XANES study, transitions occurred with the hybridization of O 2*p*_σ_ and O 2*p*_π_-derived states. These transitions/hybridizations in (ZnO: Er)_sapphire/silicon_ thin films are lower than pure ZnO due to Er-substitution with ZnO, which implies the existence of zinc/oxygen vacancies in the (ZnO: Er)_sapphire/silicon_ thin films. The Er-ions are in trivalent states in ZnO: Er thin films, and the ferromagnetism is caused by the substitution of Zn^2+^ in the ZnO lattice by Er^3+^-ions. Results shows that the magnetic moment in (ZnO: Er)_sapphire_ is higher than (ZnO: Er)_silicon_ thin film due to more Er^3+^-ions occupying adjacent cation lattice positions. With the reduction of oxygen vacancies by Er-substitution in ZnO, neighboring Er-ions coupled *via* oxygen vacancy/bonds (ferromagnetic exchange/super-exchange interaction) are coupled in (ZnO: Er)_sapphire_, which are responsible for the higher value of M_S_. The overall occupying of Er^3+^-ions due to higher vacancies/hybridization in (ZnO: Er)_sapphire_ thin film, provides the higher ferromagnetic signals at room temperature as observed in magnetic M-H hysteresis loops, where the magnetic parameters M_S_/H_C_ in (ZnO: Er)_sapphire_ films is greater than the (ZnO: Er)_silicon_ films. These (ZnO: Er)_sapphire/silicon_ thin film DMSs could be employed for optoelectronic as well as spintronic applications.

## Methods

### Preparation of ZnO and Er-substituted ZnO

ZnO and Er-ZnO thin films were grown using the DC sputtering technique using sapphire and silicon substrates, respectively. Highly dense ZnO and Er-substituted ZnO (Zn_0.95_Er_0.05_O) targets of size 2.5 cm diameter and 0.5 cm thickness were prepared by solid state reaction technique. The targets were sintered at 650 °C for 5 h to have higher densification. These targets were used in DC sputtering unit. The sputtering chamber was evacuated with high purity Ar and then O_2_ separately to remove the unwanted volatile impurities present within the chamber. The sputtering was carried out using a plasmagen gas mixture of argon and oxygen with a percentage ratio of 70:30 and the chamber pressure was kept at ~ 10^−6^ Torr. The sputtering power was fixed at maximum of 100 W. The silicon and sapphire substrates were used for depositing ZnO and Er-substituted ZnO over it. Initially the substrates were ultrasonically cleaned using acetone, isopropanol, HF and mixture of HNO_3_ & H_2_SO_4_, stepwise. Finally, the substrates were cleaned using potassium dichromate and isopropanol. Once the substrate was cleaned, it was fixed in the substrate holder present in the sputtering unit. The substrate temperature was kept at 700 °C during deposition and later annealed at the same temperature to improve the oxygen content with a heating and cooling rate of 5 °C/min.

### Characterization

The surface morphology and microstructure were investigated by scanning electron microscopy (SEM) (Philips, XL30). The crystal structure was analyzed with Rigaku Miniflex XRD in the Bragg diffraction angle of 10° to 80° using CuK_α_ radiation source (λ = 1.5404 Å) at 40 KV and 15 mA. Raman spectra were measured using HORIBA scientific XploRA at 532 nm (2.41 eV) LASER light excitation energy. Magnetic properties of ZnO: Er are typically measured using techniques like the superconducting quantum interference device (SQUID) to analyses magnetization curves. The Zn *K*-edge and Er *L*_3_-edge XANES measurements were taken at the Wiggler-17C1 beamline; whereas Zn *L*_3_ edge and O *K*-edge XANES measurements were taken at the 20A1 beamlines, and XPS measurements were taken from TLS-09A1 beamlines of Taiwan Light Source at the National Synchrotron Radiation Research Center (NSRRC) in Hsinchu, Taiwan. The Zn *K*-edge EXAFS spectra were obtained in fluorescence mode, while the O *K*-edge and Zr/Zn *L*_3_-edge spectra were obtained in surface-sensitive electron-yield mode. The resolution was set to 0.1 eV (0.2 eV) at a photon energy of 530 eV (1020 eV) for the O *K*-edge (Zn *L*_3_-edge) XANES measurements. All XANES measurements were carried out to study the electronic structures of ZnO: Er thin films deposited on sapphire and silicon substrates. Zn *K*-edge and Er *L*_3_-edge EXAFS were performed to investigate the local structure of Er in ZnO: Er thin films.

## Data Availability

All data generated or analysed during this study are included in this manuscript files.
